# Pleiotropic effects of acarbose on atherosclerosis development in rabbits are mediated via upregulating AMPK signals

**DOI:** 10.1038/srep38642

**Published:** 2016-12-07

**Authors:** Kuei-Chuan Chan, Meng-Hsun Yu, Ming-Cheng Lin, Chien-Ning Huang, Dai-Jung Chung, Yi-Ju Lee, Cheng-Hsun Wu, Chau-Jong Wang

**Affiliations:** 1 Department of Internal Medicine, Chung-Shan Medical University Hospital, No. 110, Sec. 1, Jianguo N. Road, Taichung, 402, Taiwan; 2 School of Medicine, Institute of Medicine, Chung-Shan Medical University, No. 110, Sec. 1, Jianguo N. Road, Taichung, 402, Taiwan; 3 Institute of Biochemistry, Microbiology and Immunology, Chung Shan Medical University, No. 110, Sec. 1, Jianguo N. Road, Taichung 402, Taiwan; 4 Department of Pathology, Chung Shan Medical University Hospital, School of Medicine, Chung Shan Medical University, No. 110, Sec. 1, Jianguo N. Road, Taichung, 402, Taiwan; 5 Department of Anatomy, China Medical University, Taichung, 404, Taiwan; 6 Department of Medical Research, Chung Shan Medical University Hospital, No. 110, Sec. 1, Jianguo N. Road, Taichung, 402, Taiwan

## Abstract

Acarbose, an α-glucosidase inhibitor, is reported to reduce the incidence of silent myocardial infarction and slow the progression of intima-media thickening in patients with glucose intolerance. Here we investigate other impacts of acarbose on atherosclerosis development and the underlying mechanisms of atherosclerosis initiation and progression *in vivo* and *in vitro*. Rabbits fed a high cholesterol diet (HCD) were treated with acarbose (2.5–5.0 mg kg^−1^). Immunohistochemistry was used to assess the expression of inducible nitric oxide synthase (iNOS), Ras, proliferating cell nuclear antigen (PCNA), IL-6, β-galactosidase, and p-AMPK in atherosclerotic lesions. Treatment with acarbose in HCD-fed rabbits was found to significantly reduce the severity of aortic atheroma and neointimal expression of α-actin, PCNA, IL-6, TNF-α, Ras, and β-galactosidase; to significantly increase expression of iNOS and p-AMPK, but not to affect serum levels of glucose, total cholesterol, and LDL. Western blot analysis showed acarbose dose-dependently decreased β-galactosidase and Ras expression and increased p-AMPK expression in TNF-α-treated A7r5 cells. In addition, acarbose restored p-AMPK and iNOS levels in AMPK inhibitor- and iNOS inhibitor-treated A7r5 cells, respectively. In conclusion, acarbose can pleiotropically inhibit rabbit atherosclerosis by reducing inflammation, senescence, and VSMCs proliferation/migration via upregulating AMPK signals.

Atherosclerosis is a chronic inflammatory process involving many cells (mainly monocytes/macrophages and T lymphocytes) as well as injury to vascular endothelial cells by oxidized low density lipoprotein (ox-LDL). Macrophages activated by ox-LDL secrete tumor necrosis factor alpha (TNF-α) and interleukins^1^. In the elderly, atherosclerotic cardiovascular diseases are the leading cause of morbidity and mortality^2^, and systemic chronic inflammation is related to all-cause mortality risk^3^. After undergoing a number of divisions, normal somatic cells enter a state of irreversibly arrested growth known as replicative senescence. This process is also thought to reflect aging^4^. In addition, increased inflammatory activity in the blood (including increased circulating levels of TNF-α, interleukin 6 (IL-6), and C-reactive protein) may be involved in age-associated decline. Age-related vascular cell senescence resulting in endothelial dysfunction and senescent changes in vascular cell function, morphology, and gene expression contribute to the development of atherosclerosis^5^,^6^, while abnormal vascular smooth muscle cell (VSMC) proliferation plays an important role in the pathogenesis of both atherosclerosis and restenosis after percutaneous coronary interventions^7^.

Acarbose is administered clinically to control postprandial hyperglycemia by inhibiting α-glucosidase activity in the small intestine. Postprandial hyperglycemia can induce atherosclerosis by increasing vascular inflammation, platelet activation, and oxidative stress^8^. By decreasing postprandial hyperglycemia in glucose intolerant patients, acarbose reportedly reduces the incidence of hypertension and cardiovascular events^9^ and silent myocardial infarctions^10^. Acarbose also reduces myocardial infarct size in animals by opening mitochondrial KATP channels^11^, slows progression of intima-media thickening in subjects with impaired glucose tolerance, improves carotid plaque echogenicity in patients with acute coronary syndrome^12^,^13^, and reduces the risk of myocardial infarction in type 2 diabetic patients^14^. An *in vivo* study in rabbits showed that acarbose can improve atherosclerosis by decreasing levels of aortic sudanophilia, plasma cholesterol, and plasma LDL^15^. Previous clinical trials showed acarbose treatment can reduce elevated cholesterol concentrations in non-insulin dependent diabetics and non-diabetic patients^16^,^17^. However, another clinical trial showed that in patients with non-insulin-dependent diabetes, acarbose reduces serum triglyceride levels without affecting serum cholesterol levels^18^. The anti-atherosclerotic mechanism of acarbose beyond glucose lowering is not fully understood. The present study investigated the impact of acarbose on atherosclerosis beyond its glucose-lowering effect and sought a more complete understanding of the underlying mechanisms *in vivo* and *in vitro.*

## Results

### The effect of acarbose on body weight and serum levels

To examine the effects of acarbose *in vivo*, New Zealand white rabbits were fed a high-cholesterol diet feed containing 3% lard oil + 0.5% cholesterol for 25 weeks. Consumption of the HCD for 25 weeks resulted in a significant increase in body weight gain of the rabbit. The body weight gain of the HCD group significantly increased compared with that of the control group. However, treatment with acarbose (2.5 mg kg^−1^ and 5 mg kg^−1^) did not cause any change body weight, on serum levels of triglyceride, total cholesterol, LDL, and glucose in HCD-fed rabbits (Table 1).

### Acarbose reduced intimal hyperplasia and VSMC proliferation/migration

**Figure 1:**
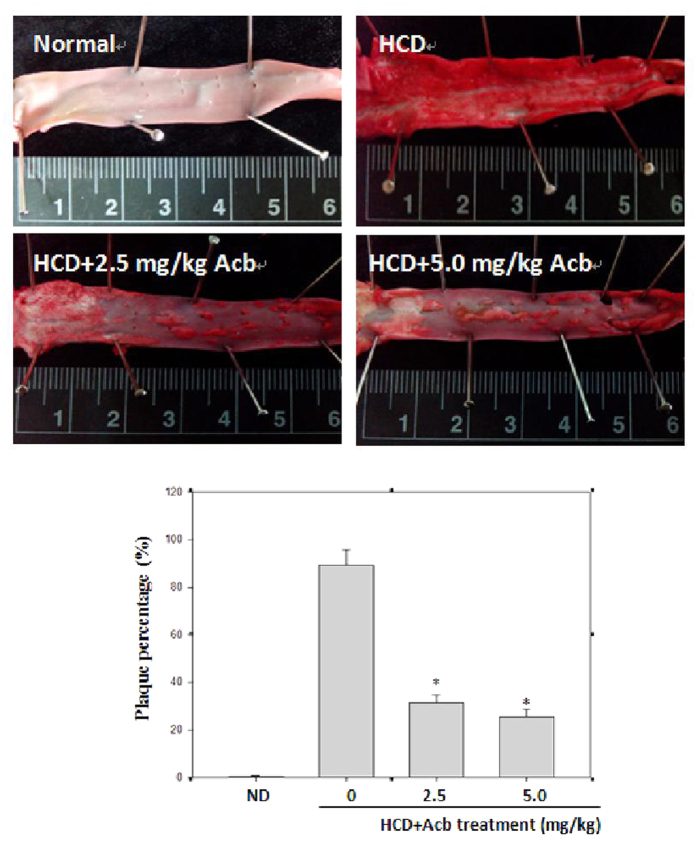
Acarbose inhibits atherosclerosis development in HCD-fed rabbits. Representative photographs showing cross sections of the aorta. Aortic arches from rabbits fed a normal diet, high cholesterol diet (HCD), HCD + Acarbose (2.5 mg kg^−1^ per day), or HCD + Acarbose (5.0 mg kg^−1^ per day) were dissected and then stained with Oil Red O. The percentage of the aortic area stained with Oil Red O was calculated. Values are shown as mean ± SD. n = 6/group. *p < 0.05, compared with the HCD group.

**Figure 2:**
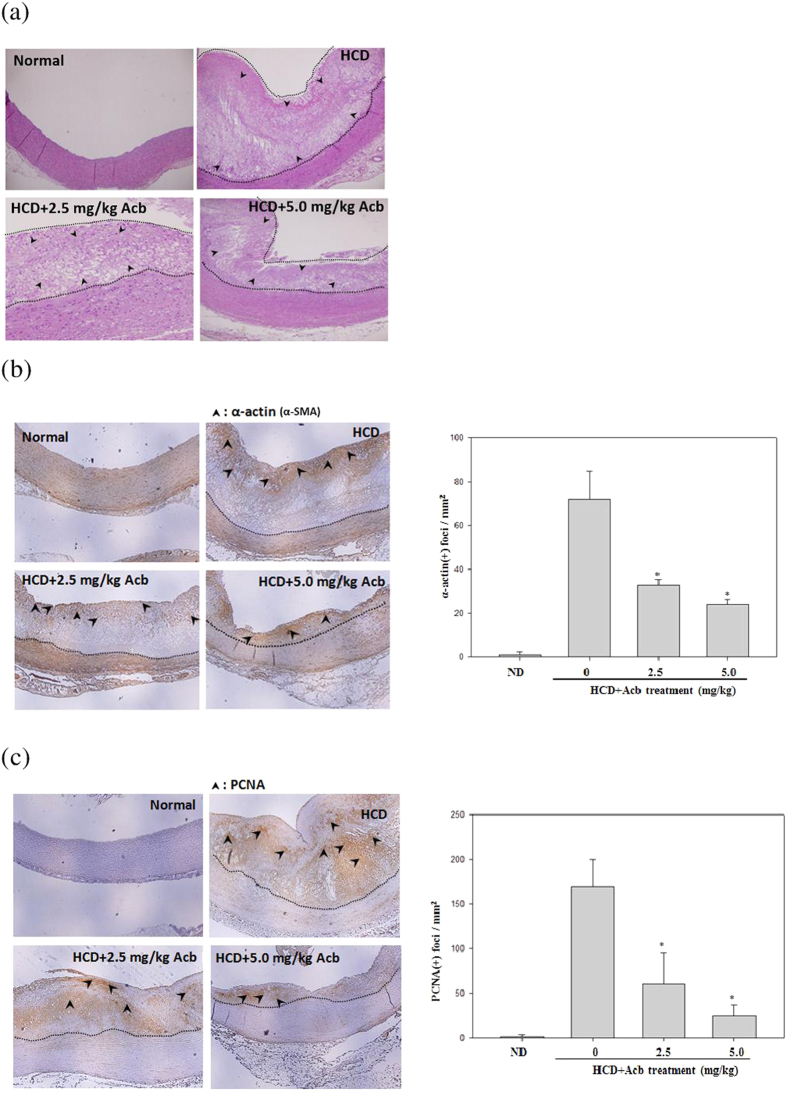
Effects of acarbose in HCD-fed rabbits on the immunostaining of α-SMA and PCNA in the neointima of atherosclerotic lesions. Photographs show cross-sections of aortic arches obtained from animals fed a normal diet, HCD, HCD + Acarbose (2.5 mg kg^−1^ per day), and HCD + Acarbose (5.0 mg kg^−1^ per day) (40X magnification). The areas of the intima and neointima are respectively indicated by arrowheads and dotted lines. The luminal surface of the aortic arch after (**a**) H&E staining (indicated by arrowheads and dotted lines) and (**b,c**) immunostaining of (**b**) α-SMA and (**c**) PCNA (indicated by arrowheads). (**a**–**c**) Cells are proliferating in the aortic segments from rabbits fed the HCD. The area of atherosclerotic foci was determined in five randomly selected fields (0.2 mm^2^ each) in three aortic segments from each group using the Image-Pro Plus analysis system. Values are shown as mean ± SD. n = 6/group. *p < 0.05, compared with the HCD group.

**Figure 3:**
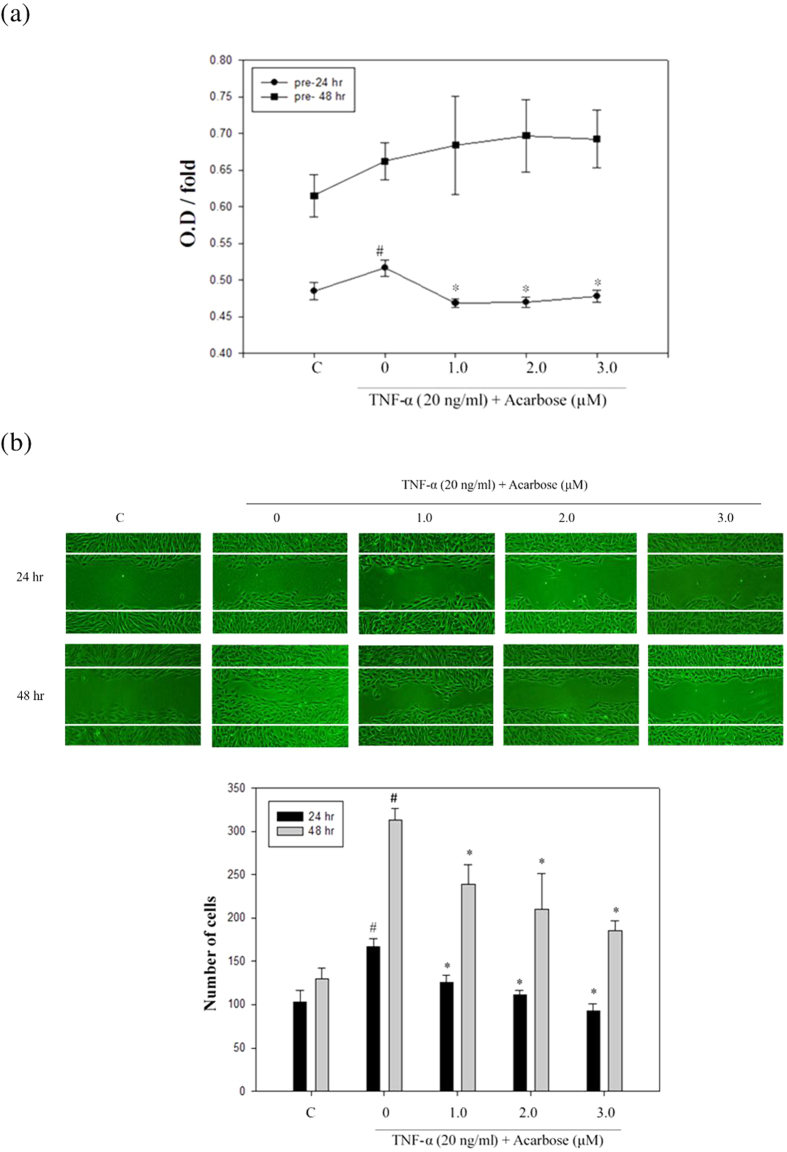
Effect of acarbose on TNF-α induced proliferation and migration in A7r5 cells (VSMCs). (**a**) A7r5 cells were pre-treated with TNF-α (20 ng/ml) and then co-treated with acarbose (0, 1, 2, or 3 μM) for the indicated times (24 and 48 h). Cell viability was analyzed by the MTT assay. Data are presented as the mean ± SD (n = 3). (**b**) The wound healing assay was performed on cell monolayers treated with TNF-α (20 ng/ml) and then co-treated with acarbose (0, 1, 2, or 3 μM) for 24 and 48 h. Treatment with acarbose decreased the migration of A7r5 cells. The mean number of cells was determined at 24 and 48 h in the denuded zone and represents the average of 3 independent experiments ± SD. ^#^p < 0.05, as compared with the control group. *p < 0.05, as compared with the TNF-α alone group.

Athersclerosis is in part characterized by migration of smooth muscle cells from media to intima and proliferation. To determine whether acarbose acts on VSMC proliferation and migration, sections of aortic arch were immunostained for smooth muscle α-actin and PCNA. The numbers of atherosclerotic plaques in the aortic arch were significantly increased in HCD-fed rabbits compared with normal-diet-fed rabbits and significantly decreased in the HCD group by acarbose treatment (Fig. 1). In the HCD group, H&E staining revealed marked intimal hyperplasia and pronounced regression of intimal hyperplasia after the acarbose treatment (Fig. 2a), and immunostaining for smooth muscle α-actin (α-SMA) and PCNA (Fig. 2b,c) showed significant and dose-dependent decreases in neointimal levels of these two markers after treatment with acarbose (2.5 and 5.0 mg kg^−1^). To further confirm the impact of acarbose on proliferation and migration of VSMCs, we exposed TNF-α-treated A7r5 cells to non-toxic concentrations of acarbose (1, 2, and 3 μM) and showed that acarbose dose- and time-dependently inhibited TNF-α-induced VSMC proliferation and migration (Fig. 3).

### Acarbose reduced HCD-induced inflammation

**Figure 4:**
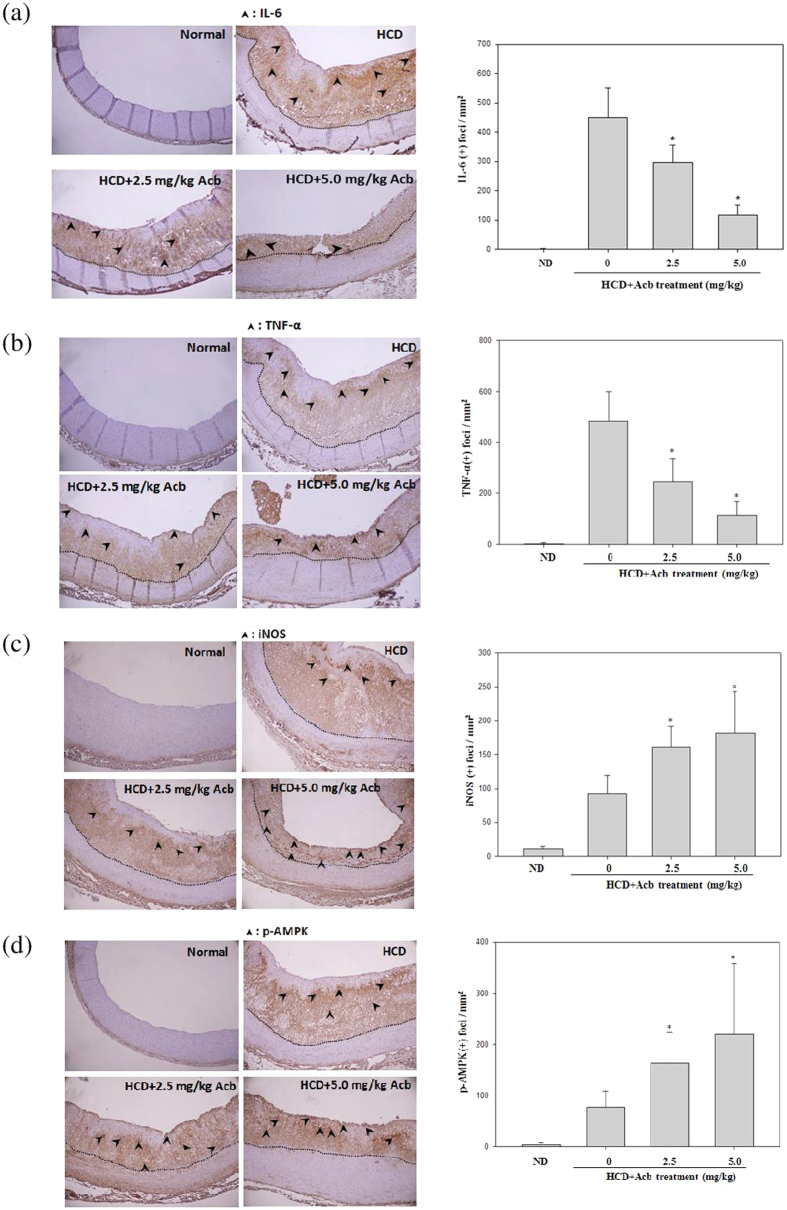
Effects of acarbose in HCD-fed rabbits on immunostaining of IL-6, TNF-α, and iNOS in the neointima of atherosclerotic lesions. Photographs show cross-sections of aortic arches obtained from animals fed a normal diet, HCD, HCD + Acarbose (2.5 mg kg^−1^ per day), and HCD + Acarbose (5.0 mg kg^−1^ per day) (40X magnification). The areas of the intima and neointima are respectively indicated by arrowheads and dotted lines. The luminal surface of the aortic arch after immunostaining of inflammation-related proteins (**a**) IL-6; (**b**) tumor necrosis factor–alpha (TNF-α); (**c**) iNOS, and (**d**) p-AMPK is shown. The area of atherosclerotic foci was determined in five randomly selected fields (0.2 mm^2^ each) in three aortic segments from each group using the Image-Pro Plus analysis system. Values are shown as mean ± SD. n = 6/group. *p < 0.05, compared with the HCD group.

To evaluate the role of acarbose in HCD-induced inflammation, we used immunohistochemistry to analyze the effect of acarbose on the levels of IL-6, TNF-α, and iNOS and the metabolism-related protein kinases (p-AMPK), all of which are elevated by HCDs. Acarbose (2.5 and 5.0 mg kg^−1^) significantly and dose-dependently decreased the intensity of neointimal IL-6 (Fig. 4a), TNF-α (Fig. 4b), and iNOS (Fig. 4c) staining, and significantly increased the intensity of neointimal p-AMPK staining (Fig. 4d).

### Acarbose reduced HCD-induced aging

**Figure 5:**
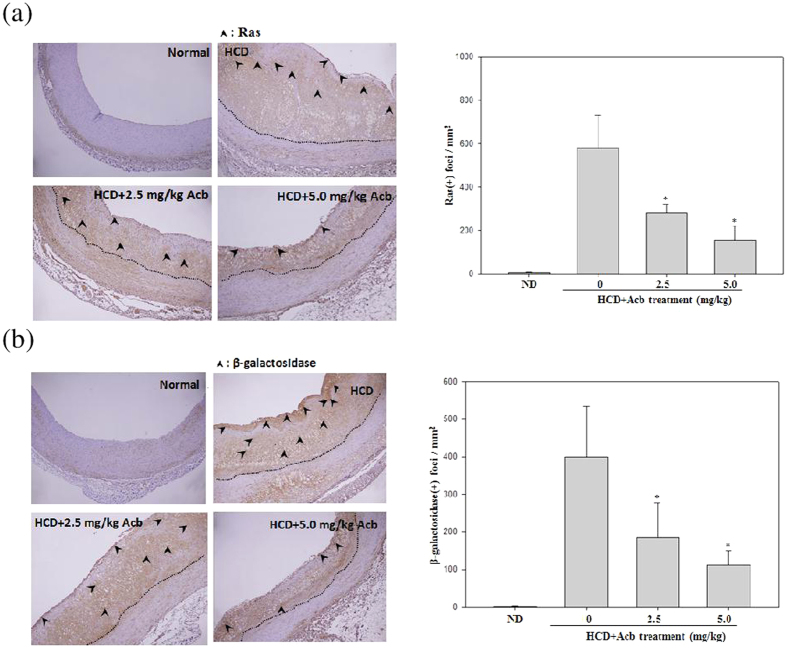
Effects of acarbose in HCD-fed rabbits on immunostaining of Ras and β-galactosidase in the neointima of atherosclerotic lesions. Photographs show cross-sections of aortic arches obtained from animals fed a normal diet, HCD, HCD + Acarbose (2.5 mg kg^−1^ per day), and HCD + Acarbose (5.0 mg kg^−1^ per day) (40X magnification). The areas of the intima and neointima are respectively indicated by arrowheads and dotted lines. The luminal surface of the aortic arch after immunostaining of the proliferation-, migration-, and aging-associated proteins (**a**) Ras and (**b**) β-galactosidase is shown. The area of atherosclerotic foci was determined in five randomly selected fields (0.2 mm^2^ each) in three aortic segments from each group using the Image-Pro Plus analysis system. Values are shown as mean ± SD. n = 6/group. *p < 0.05, compared with the HCD group.

**Figure 6:**
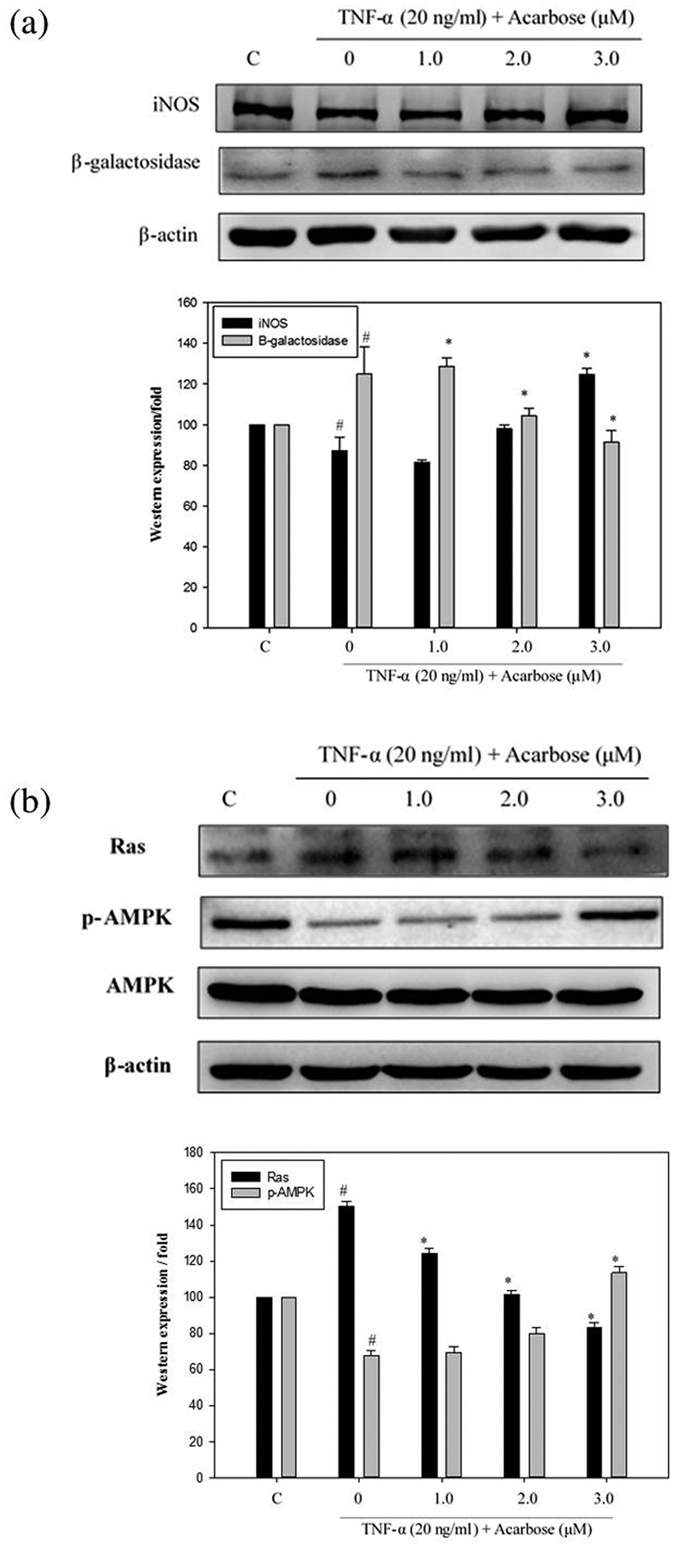
Effect of acarbose on the expressions of iNOS and β-galactosidase in A7r5 cells pre-treated with TNF-α (20 ng/ml) for 24 h. Subsequently, acarbose (1.0, 2.0, or 3.0 μM) was added for 24 h. The cell lysates for protein extraction were prepared and the total protein of each lysate was equalized for Western blot analysis. Proteins were detected by specific antibodies to (**a**) iNOS and β-galactosidase, (**b**) p-AMPK and Ras. β-actin was used as a loading control. Each mean value is the average of 3 independent experiments ± SD. ^#^*p* < 0.05, as compared with control group. *p < 0.05, as compared with the TNF-α alone group.

Evidence suggests that cellular senescence is involved in the atherosclerotic process. Levels of aging-related proteins such as Ras and β-galactosidase were determined to assess the influence of acarbose on HCD-induced aging. Acarbose (2.5 and 5.0 mg kg^−1^) significantly and dose-dependently decreased neointimal Ras and β-galactosidase expression in HCD-fed rabbits (numbers of neointimal Ras and β-galactosidase foci; Fig. 5a and b) and acarbose (1, 2, and 3 μM) dose-dependently decreased β-galactosidase, Ras expression and increased p-AMPK expression in TNF-α pre-treated A7r5 cells (Western blot; Fig. 6a and b).

### Effect of acarbose on the proliferation-related protein

In previous figures, iNOS and p-AMPK levels increased in IHC stain of acarbose-treated HCD-fed rabbits. Therefore we further focus on the expression of iNOS and p-AMPK by western blotting after TNF-a pre-treated A7r5 cells treated with or without acarbose (1–3 μM). The data shows that the protein levels of iNOS and p-AMPK were increased in acarbose dose-dependent.

**Figure 7:**
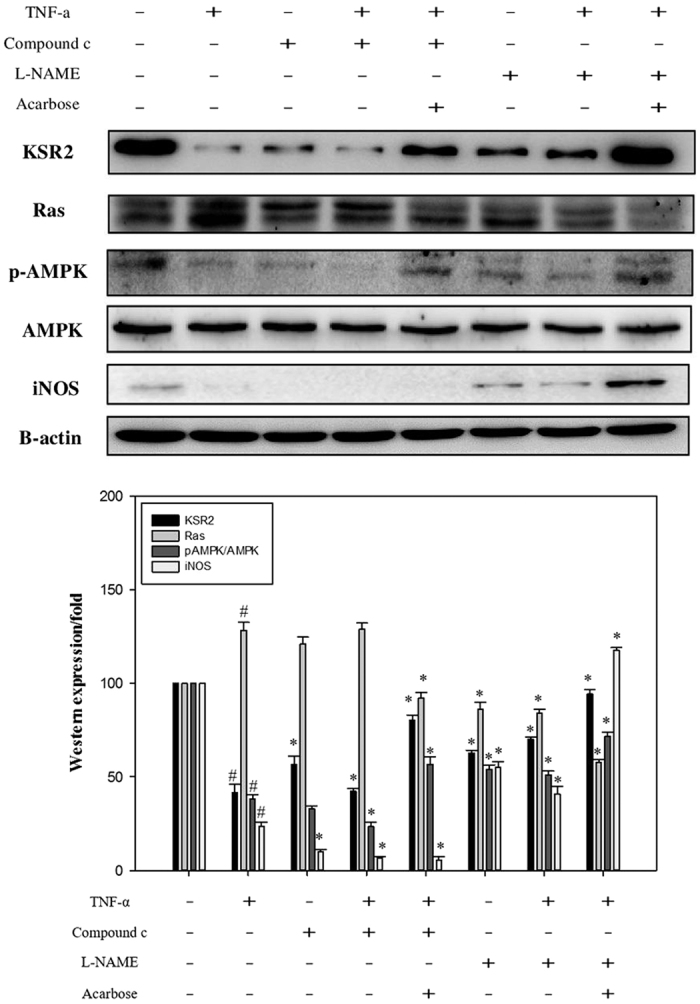
Effect of inhibitors on the expressions of KSR2, Ras, p-AMPK, and iNOS in A7r5 cells pre-treated with TNF-α (20 ng/ml) for 24 h. Subsequently, acarbose (3.0 μM) was added for 24 h in the absence and presence of 5 μM AMPK inhibitor or 0.5 mM L-NAME for 30 min. The cell lysates for protein extraction were prepared and the total protein of each lysate was equalized for Western blot analysis. Proteins were detected by specific antibodies to KSR2, Ras, p-AMPK, and iNOS. β-actin was used as a loading control. ^#^*p* < 0.05, as compared with control group. *p < 0.05, as compared with the TNF-α alone group.

Next, compound C (inhibitor of AMPK) and L-NAME (inhibitor of iNOS) were used to examine p-AMPK and iNOS activation by acarbose. Indeed, acarbose enhanced p-AMPK and iNOS expression in the presence of their respective inhibitors (Western blot: Fig. 7), suggesting that the acarbose-induced increase in p-AMPK expression affects migration and proliferation.

## Discussion

Recent studies have shown that treating IGT (Impaired glucose tolerance) patients with acarbose is associated with a significant reduction in the risk of cardiovascular disease and hypertension^19^. However, acarbose specific moderate glucose level in IGT situation. Our study demonstrated that acarbose can effectively reduce atheroma progression in HCD-fed rabbits without affecting their body weight, serum levels of triglyceride, total cholesterol, LDL, and glucose. Therefore, acarbose acts pleiotropically to suppress atherosclerosis not involving glucose and LDL reduction.

PCNA expression is increased in unstable atherosclerotic carotid plaque^20^. Moreover, the expression of PCNA is expressed in human vascular smooth muscle cell^21^. Our study showed a significant and dose-dependent decrease in neointimal expression of PCNA after treatment with acarbose (2.5 and 5.0 mg kg^−1^) in HCD-fed rabbits, indicating that acarbose ameliorates atherosclerosis by reducing PCNA expression.

The inflammatory cytokine TNF-α reportedly plays a vital role in the disruption of the macrovascular and microvascular circulation and its increased expression induces the production of reactive oxygen species, resulting in endothelial cell damage and dysfunction^22^. High blood levels of TNF-α have been associated with a high prevalence of atherosclerosis and dementia^5^,^23^ and blockade of TNF-α may improve cardiovascular morbidity and mortality in chronic inflammatory disease^24^. Circulating levels of TNF-α and IL-6 are increased in healthy elderly individuals and patients with type 2 diabetes^25^. Systemic inflammation, as measured by IL-6, may be associated with future cardiovascular events and the clinical evolution of cardiovascular disease in older patients^26^. NO, which is synthesized by NOS, is not only the most potent vasodilator, but also an inhibitor of VSMC proliferation and platelet adherence and aggregation^27^,^28^. Many disorders (including hypercholesterolemia, diabetes mellitus, hypertension, and smoking) are associated with reduced synthesis of vascular NO. Reduced NO release leads to the development of atherosclerosis^28^. In our study, treatment with acarbose (2.5 and 5.0 mg kg^−1^) led to a significant and dose-dependent increase in neointimal IL-6 and TNF-α expression and decrease in neointimal iNOS expression in HCD-fed rabbits, indicating that acarbose inhibits atherosclerosis by reducing IL-6- and TNF-α-associated chronic inflammation and by increasing NO.

Through activation of extracellular signal-regulated kinase (ERK), Ras can induce VSMC senescence and vascular inflammation in human atherosclerosis, thus providing a new anti-senescence target for atherosclerosis treatment^6^. Several human cells reportedly express β-galactosidase upon senescence^4^, and expression of β-galactosidase during the replicative senescence of human endothelial cells reportedly reflects an increase in lysosomal volume^29^. Our finding of significant and dose-dependent decrease in neointimal Ras and β-galactosidase expression after treatment with acarbose (2.5 and 5.0 mg kg^−1^) in HCD-fed rabbits indicated that acarbose inhibits senescence and atherosclerosis by also reducing Ras and β-galactosidase expression.

AMPK reportedly improves endothelial function, attenuates myocardial ischemia, inhibits human VSMC proliferation, and suppresses neointimal formation after balloon angioplasty^30^,^31^,^32^. Increased levels of reactive oxygen species (ROS), often associated with cardiovascular disease in the elderly, can decrease NOS activation and NO synthesis via activation of AMPK. Induction of the AMPK-NOS pathway has a protective role in endothelial homeostasis^33^. Gallic acid has been shown to attenuate cell cycle progression via AMPK-mediated NOS activation, thus preventing atherosclerosis^34^. AMPK might also slow aging by increasing nitric oxide synthesis and protecting vascular endothelial function^35^. The Ras/Raf/MEK/ERK pathway regulates cell proliferation, differentiation, survival, and apoptosis^36^ and its activation along with AMPK inhibition has been associated with the aging process^37^. iNOS mechanisms are required for VSMC proliferation in response to TNF-α. However, Haider *et al*.^38^ have demonstrated the dual functionality of NO-mediated inhibition of VSMC proliferation. In the presence of TNF-α, VSMCs overcome the inhibitory influence of NO on proliferation. On the other hand, iNOS contributes to the anti-proliferative effect of NO in the absence of TNF-α. In our study, the anti-inflammatory effect of acarbose enhanced NO expression to arrest cell cycle progression and inhibit VSMC proliferation. We proposed that acarbose increases iNOS and NO production through activation of AMPK and inhibition of Ras, thus preventing atherosclerosis and slowing development of atherosclerosis.

Because our IHC data indicated that HCD can effectively promote atherosclerosis in rabbits via inducing TNF-α secretion, we further used TNF-α to induce proliferation and migration of VSMCs and then treated them with acarbose to confirm the effect of acarbose on atherosclerosis. The acarbose-treatment group appeared that proliferation and migration relative protein decline significantly both *in vitro* and *in vivo*.

**Figure 8:**
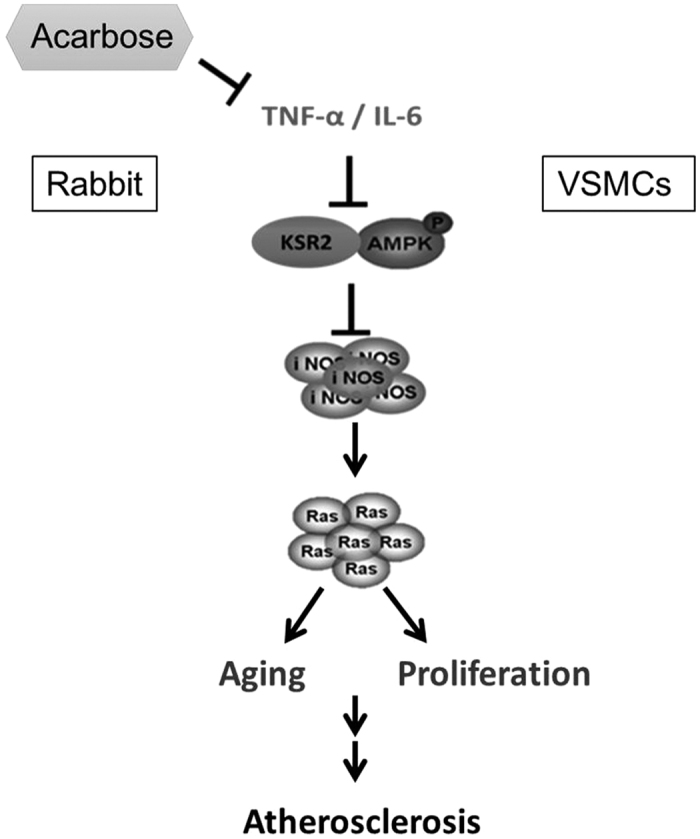
Schematic diagram of the proposed mechanism of acarbose-induced inhibition of atherosclerosis in rabbits and VSMC proliferation/migration via the AMPK-NO-Ras signaling pathway.

In summary, the inhibition of atherosclerosis development via inhibition inflammation and senescence of VSMCs in rabbit, therefore the proliferation and migration situation could be retarded. The above-mentioned mechanism involves regulation of the AMPK-NO-Ras signaling pathway (Fig. 8). Above all, acarbose could improve vascular inflammation and senescence of VSMCs via adjustion of the AMPK-NO-Ras signaling pathway.

## Materials and Methods

### Animals and diets

Twenty-four male New Zealand white rabbits (Animal Center of Chung Shan Medical University), weighing 2500 g were used. They were individually housed in metal cages in an air-conditioned room (22 ± 2 °C, 55 ± 5% humidity), under a 12 h light/12 h dark cycle with free access to food and water. All rabbits were randomly assigned to four groups of 6 animals each and were fed either standard chow (Group I), high cholesterol diet (HCD; containing 95.7% standard Purina chow + 3% lard oil + 0.5% cholesterol) (Group II), HCD diet and 2.5 mg kg^−1^ per day acarbose (Group III), or HCD diet and 5.0 mg kg^−1^ per day acarbose (Group IV). During the 25-week feeding period, the handling of animals followed the guidelines of the Institutional Animal Care and Use Committee of Chung Shan Medical University (IACUC, CSMC). At the end of the 25 weeks, all rabbits were sacrificed by exsanguination under deep anesthesia with pentobarbital (30 mg kg^−1^ i.v.) injected via the marginal ear vein. Serum was stored at −80 °C prior to measurement of serum values. The aortic arch and thoracic aortas were carefully removed to protect the endothelial lining, and were collected and freed of adhering soft tissue. All animal care and experimental procedures were carried out in strict accordance with the guidelines for the care and use of laboratory animals of Chung Shan Medical University, and approved by the Institutional Animal Care and Use Committee. This article does not contain clinical studies or patient data.

### Blood sample analysis

Blood samples were collected and centrifuged at 1500 *g* for 10 min at 4 °C. The serum was decanted and stored at −20 °C. Total serum cholesterol level was measured using reaction buffer (0.3 mM 4-aminoantipyrine, 6 mM phenol, 0.5 U mL^−1^ peroxidase, 0.15 U mL^−1^ cholesterol esterase, and 0.1 U mL^−1^ cholesterol oxidase) and a colorimetric method (λ = 500 nm)^39^. The plasma for LDL-C measurement was obtained from blood collected in tubes containing 5000 IU L^−1^ heparin and 0.064 M sodium citrate and incubated at room temperature for 10 minutes before centrifugation at 4000 rpm for 15 minutes. Supernatant plasma (50 μL) was mixed with phosphate buffer solution (PBS) containing 0.3 mM 4-aminoantipyrine, 6 mM phenol, 0.5 U mL^−1^ peroxidase, 0.15 U mL^−1^ cholesterol esterase, and 0.1 U mL^−1^ cholesterol oxidase, and the mixture was incubated at room temperature for 10 minutes and assayed using a colorimetric method (λ = 500 nm)^40^. Serum glucose was measured colorimetrically using an automatic analyzer (Olympus AU2700, Olympus Co., Tokyo, Japan)^41^.

### Evaluation of atherosclerotic lesions

The aortic arches were rapidly dissected and fixed in 10% neutral-buffer formalin (NBF). The sections of aortic arch were stained with hematoxylin and eosin (H&E) or α-SMA (smooth muscle actin) antibodies (Santa Cruz Biotechnology, Inc., Santa Cruz, CA, USA). Immunohistochemistry was carried out to confirm the presence of atherosclerotic lesions. The immunohistochemical results were evaluated independently by experienced pathologists.

### Immunohistochemistry (IHC)

Briefly, paraffin-embedded sections (10-μm thick) were deparaffinized, treated with 3% H_2_O_2_ in methanol for 10 min to inactivate any endogenous peroxidase, washed with PBST (0.1% Tween 20 in PBS), incubated for 60 min in blocking buffer (3% bovine serum albumin [BSA] in PBST), incubated for 60 min with primary antibodies to inducible nitric oxide synthase (iNOS), Ras (Santa Cruz Biotechnology), α-actin (Sigma, St. Louis, MO, USA), proliferating cell nuclear antigen (PCNA) (Dako Cytomation, Carpinteria, CA, USA), IL-6, TNF-α, β-galactosidase, (Abcam PLC, Cambridge, UK), and adenosine 5′-monophosphate-activated protein kinase (p-AMPK; Cell Signaling, Beverly, MA, USA), all diluted in 1% BSA, washed three times for 10 min in PBST, incubated 60 min with the horseradish peroxidase (HRP)-conjugated secondary antibody (Sigma) diluted in 1% BSA at 37 °C, and finally incubated 3 min at room temperature with 3, 3′-diaminobenzidine (DAB) for color development. All experiments were repeated three times.

### Image-Pro Plus analysis

The images of immunostained rabbit thoracic aortas were analyzed using Image Pro-Plus (IPP) software (Media Cybernetics, Silver Spring, MD, USA) to calculate the density mean, area sum, and integrated optical density (IOD) of positive expression, which was compared with visually assessed staining intensity and percentage of stained cells. The IPP analysis system was used to first create and measure 0.2-mm^2^ areas of interest (AOIs) in five randomly selected fields of the acquired image from three tissues of every group, measure the optical density in each AOI, and subtract the background optical density^42^,^43^.

### Cell culture

The rat thoracic aorta smooth muscle cell line A7r5 was purchased from Bioresource Collection and Research Center (BCRC) (BCRC number: 60082). A7r5 cell was cultured in Dulbecco’s modified Eagle’s medium (DMEM) (Gibco) supplemented with 10% fetal bovine serum (FBS), 1% L-glutamine, 1% g/L sodium bicarbonate and 1% penicillin/streptomycin (Hyclone). All cultures were maintained in a humidified 5% CO2 atmosphere at 37 °C. Before treatment, the A7r5 cell was precultured in 0.5% FBS medium for 48 hr.

### Cell viability analysis

Cell viability was determined using the 3-(4,5-dimethylthiazol-2-yl)-2,5- diphenyltetrazolium bromide (MTT) assay^44^. Cells were seeded in 24-well culture plates at a density of 2 × 10^4^ cells/well, incubated for 48 h, treated with acarbose at varying concentrations (0.5, 1.0, 2.0, 3.0, and 5.0 μM) for 24 h; or pre-treated with TNF-α (20 ng/ml) for either 24 h or 48 h to evaluate the dose-dependent effects of acarbose on VSMC growth and viability, cultured with 0.5 mg/ml MTT at 37 °C in a humidified atmosphere of 5% CO_2_ for another 4 h, and solubilized with isopropanol. The viable cell number varied directly with the concentration of formazan product measured spectrophotometrically at 563 nm.

### Wound healing

A7r5 cells were seeded at a density of 1 × 10^6^ ml in 6-well culture plates and incubated for 48 h. A sterile 100-μl pipette tip was used to make a straight scratch in the cell monolayer in each well^45^. The non-adhering cells were washed out with PBS, and the remaining cells were treated with TNF-α (0, 10, 20, 50 and 100 ng/ml) at 37 °C in a humidified atmosphere of 5% CO_2_. Under a 40X lens, images of the linear wound (9 fields per well) were taken at 24 and 48 h. Migrated cells were counted per well and the counts were averaged.

### Western blot analysis

Western blot analysis^46^ was used to assess the expressions and/or activities of these migration-related proteins and thereby the mechanisms underlying the anti-migratory effects of acarbose on VSMCs. Specific antibodies were used to evaluate the expressions of iNOS (Santa Cruz Biotechnology), Ras (Santa Cruz Biotechnology), p-AMPK (Cell Signaling), AMPKα1/2 (Santa Cruz Biotechnology), and TNF-α and β-galactosidase (Abcam). After pre-treatment with TNF-α (20 ng/ml) for 24 h, the cells were treated with acarbose (0, 1, 2, and 3 μM) for 24 h and lysed. Cell lysates (50 μg of protein) were separated by electrophoresis on 8–12% SDS polyacrylamide gels and transferred to nitrocellulose membranes (Millipore, Bedford, MA, USA). The membranes were incubated with Tris-buffered saline (TBS) containing 1% (w/v) nonfat-milk and 0.1% (v/v) Tween-20 (TBST) for 1 h to block non-specific binding, washed with TBST for 30 min, incubated with the appropriate primary antibody for 2 h, incubated with horseradish peroxidase-conjugated second antibody (Sigma) for 1 h, developed using ECL chemiluminescence (Millipore), and analyzed by densitometry using AlphaImager Series 2200 software. Compound C (an AMPK inhibitor, 5 μM) and L-NAME (iNOS inhibitor, 0.5 mM) were used to confirm AMPK and iNOS expression in TNF-α-pretreated acarbose-treated (1, 2 and 3 μM for 24 h) cells. Results are representative of at least 3 independent experiments.

### Statistical analysis

The data are presented as the mean ± standard deviation of three independent experiments and evaluated by one-way analysis of variance (ANOVA). Significant differences were established at *p* < 0.05.

## Additional Information

**How to cite this article**: Chan, K.-C. *et al*. Pleiotropic effects of acarbose on atherosclerosis development in rabbits are mediated via upregulating AMPK signals. *Sci. Rep.* **6**, 38642; doi: 10.1038/srep38642 (2016).

**Publisher's note:** Springer Nature remains neutral with regard to jurisdictional claims in published maps and institutional affiliations.

## Figures and Tables

**Table 1:** A comparison of body weight and serum levels in HCD-fed rabbits.

Treatment		Weight of rabbit (kg/rabbit)	Parameters of plasma			
HCD	Acarbose (mg)		Triglyceride (mg/dL)	Total cholesterol (mg/dL)	LDL-C (mg/dL)	Glcuose (mg/dL)
−	−	2.65 ± 0.36	58.6 ± 17.1	45.2 ± 9.5	15.2 ± 2.1	138 ± 3.05
+	−	2.91 ± 0.49*	261.2 ± 21.4*	1350 ± 52*	584.5 ± 187*	142 ± 2.08
+	2.5	2.8 ± 0.14	270.5 ± 28.2	1402 ± 57	602.4 ± 101	143 ± 4.51
+	5.0	2.79 ± 0.28	265.9 ± 25.4	1386 ± 49	598 ± 92	150 ± 2.52

Each value is expressed as the mean ± SD (n = 6 per group).

Statistical significance was analyzed with ANOVA. *p < 0.05 as compared to the control group.
